# Neonatal local noxious insult affects gene expression in the spinal dorsal horn of adult rats

**DOI:** 10.1186/1744-8069-1-27

**Published:** 2005-09-22

**Authors:** Ke Ren, Svetlana I Novikova, Fang He, Ronald Dubner, Michael S Lidow

**Affiliations:** 1 Department of Biomedical Sciences, and Program in Neuroscience, University of Maryland, Baltimore, MD 21201; USA

**Keywords:** microarray, real-time RT-PCR, neonatal injury, carrageenan, pain, development

## Abstract

Neonatal noxious insult produces a long-term effect on pain processing in adults. Rats subjected to carrageenan (CAR) injection in one hindpaw within the sensitive period develop bilateral hypoalgesia as adults. In the same rats, inflammation of the hindpaw, which was the site of the neonatal injury, induces a localized enhanced hyperalgesia limited to this paw. To gain an insight into the long-term molecular changes involved in the above-described long-term nociceptive effects of neonatal noxious insult at the spinal level, we performed DNA microarray analysis (using microarrays containing oligo-probes for 205 genes encoding receptors and transporters for glutamate, GABA, and amine neurotransmitters, precursors and receptors for neuropeptides, and neurotrophins, cytokines and their receptors) to compare gene expression profiles in the lumbar spinal dorsal horn (LDH) of adult (P60) male rats that received neonatal CAR treatment within (at postnatal day 3; P3) and outside (at postnatal 12; P12) of the sensitive period. The data were obtained both without inflammation (at baseline) and during complete Freund's adjuvant induced inflammation of the neonatally injured paw. The observed changes were verified by real-time RT-PCR. This study revealed significant basal and inflammation-associated aberrations in the expression of multiple genes in the LDH of adult animals receiving CAR injection at P3 as compared to their expression levels in the LDH of animals receiving either no injections or CAR injection at P12. In particular, at baseline, twelve genes (representing GABA, serotonin, adenosine, neuropeptide Y, cholecystokinin, opioid, tachykinin and interleukin systems) were up-regulated in the bilateral LDH of the former animals. The baseline condition in these animals was also characterized by up-regulation of seven genes (encoding members of GABA, cholecystokinin, histamine, serotonin, and neurotensin systems) in the LDH ipsilateral to the neonatally-injured paw. The largest aberration in gene expression, however, was observed during inflammation of the neonatally injured hindpaws in the ipsilateral LDH, which included thirty-six genes (encoding numerous members of glutamate, serotonin, GABA, calcitonin gene-related peptide, neurotrophin, and interleukin systems). These findings suggest that changes in gene expression may be involved in the long-term nociceptive effects of neonatal noxious insult at the spinal level.

## Introduction

Emerging evidence indicates that neonatal noxious insults result in changes in pain responsivity in adults (reviewed in: [1-3]). It has been demonstrated that these changes may, at least in part, be associated with permanent alterations in pain processing in the spinal cord [4-7].

Recently, we have developed a carrageenan (CAR) injection-based rat model of short-lasting (24 – 48 h) locally-induced noxious insult. In this model, rats subjected to a single subcutaneous injection of CAR into a hindpaw after birth, develop a long-term widespread bilateral increase in baseline nociceptive threshold along with a long-term enhanced localized ipsilateral increase in nocifensive responses (enhanced hyperalgesia) to adult inflammation of the neonatally-injured paw [8,9]. We also determined that the sensitive period for generating these effects is within the first postnatal week [9]. The present study was aimed at screening for changes in gene expression, which may be involved in the above-described long-term nociceptive effects of neonatal noxious insult at the spinal level. Here, we largely focused on three aspects of gene expression in the lumbar region of the spinal dorsal horn (LDH) of adult rats receiving CAR injection during the neonatal sensitive period. First, in expectation of gaining insight into the spinal contribution to the bilateral baseline hypoalgesia characteristic of our model, we identified changes in gene expression encompassing LDH on both ipsilateral and contralateral sides to the neonatally injured hindpaw. Second, we identified baseline changes in gene expression unique to the LDH ipsilateral to the site of neonatal injury. The interest in the latter changes was prompted by findings in several studies [4,5,7] that the apparent similarity in the latency of withdrawal responses to acute noxious stimulation in both hindpaws of adult animals, which received neonatal injections of inflammatory agents, occurs in the presence of long-term alterations in the receptive field sizes, background activity, and evoked responses of the LDH neurons receiving information from the neonatally-injured paw. Therefore, we hoped that our observations might aid in explaining why these diverse structural/physiological changes do not result in locally-altered behavioral pain responsivity. Third, we investigated abnormalities in gene regulation, which may be associated with hyperalgesia seen in our model upon adult inflammation of the neonatally-injured paw.

To achieve our goal we employed microarray profiling of gene expression in the ipsilateral and contralateral LDH of adult rats receiving neonatal CAR hindpaw injections either within or outside of the sensitive period, both at baseline and during complete Freund's adjuvant (CFA)-induced inflammation of neonatally CAR-injected paws. The analysis employed custom-designed glass microarrays containing 205 sixty-mer oligo-probes for all known genes for: glutamate, gamma-aminobutyric acid (GABA), adrenaline and serotonin transporters; glutamic acid decarboxylase (GAD); N-methyl-D-aspartate (NMDA), alpha-amino-3-hydroxy-5-methylisoxazole-4-propionic acid (AMPA), kainate, GABA_A _receptor subunits, glutamate metabotropic, GABA_B_, adenosine, adrenergic, serotonergic, histamine, and σ receptors; and peptide precursors and receptors for opioids, somatostatin (SOM), neuropeptide Y (NPY), tachykinins, neurotensin, calcitonin gene-related peptide (CGRP), cholecystokinin (CCK), and vasoactive intestinal peptide (VIP). Probes for a range of neurotrophins and cytokines and their receptors were also included. Each microarray-identified change in gene expression was verified by quantitative real-time reverse transcription-polymerase chain reactions (real-time RT-PCRs).

## Results

### Microarray-based Gene Expression Profiling

In assessing the microarray-generated data, we began by looking at the results of comparison of gene expressions in the adult (60-days-old; P60) male rats receiving a single CAR injection within the sensitive period (on P3 [9]; CAR-P3 group) and rats receiving CAR injection outside the sensitive period (on P12 [9]; CAR-P12 group) with those in the animals receiving no such injections (CONT group). Then, we turned to comparison of gene expressions between CAR-P3 and CAR-P12 rats to identify changes that may be specifically related to the CAR injection at P3. The latter comparison was aimed at discarding changes that might be common to both CAR treatments within and outside of the sensitive period.

#### Baseline Levels of Gene Expression

The search for bilateral effects identified seven genes (coding for GABA_B_1d receptor, 5-HT5a serotonergic receptor, A1 adenosine receptor, σ receptor, NPYR6 receptor, preproenkephalin, and γ-tachykinin) with significantly increased expression in the ipsilateral and contralateral LDH of CAR-P3 rats as compared to those in CONT animals (Table 1). These increases ranged from 1.5 to 3.5 fold. No significant alterations in the expression of these genes were detected in the CAR-P12 group (Table 1), which made the observed changes specific for CAR-P3 animals. Six more genes (coding for GAD65, α2b and β3 adrenergic receptors, δ-preprotachykinin, CCKR-1 receptor, and interleukin IL-10) showed higher bilateral levels of gene expression in both CAR-3 and CAR-P12 groups (Table 1). However, in CAR-P3 rats these genes were expressed at significantly higher levels (by 1.2 – 2.6 fold) than in CAR-P12 animals (Table 1). Finally, two genes (NR2B subunit of NMDA receptor and SstR2 somatostatin receptor), while being up-regulated in both CAR-P3 and CAR-P12 groups relative to CONT group, showed significantly lower levels of expression (by 0.5 and 0.7 fold respectively) in CAR-P3 rats as compared to those in CAR-P12 animals (Table 1).

**Table 1:** Genes with significantly altered basal bilateral levels in the LDH of CAR-P3 as compared to their bilateral expressions in the LDH of CONT and CAR-P12 animals [one-way ANOVAs, (with the Benjamini and Hochberg's FDR correction procedure set at the *P*-value cutoff of 0.05) followed by Tukey's post-hoc tests (with the same *P*-cutoff value) in the implementation of GeneSpring GX software (Agilent, Palo Alto, CA).)]. Differences in gene expression are presented as ratios between CAR-P3 and CONT, CARP-12 and CONT, and CAR-P3 and CAR-P12 groups. Statistically insignificant differences are marked as 'ND' The comparisons are based on the microarray profiling that employed 5 slides for comparison of CONT and CAR-P3 samples and another 5 slides for comparison of CONT and CAR-P12 samples.

**Genes**	**Left LDH (ipsilateral to the site of neonatal injury)**			**Right LDH (contralateral to the site of neonatal injury)**		
	
	CAR-P3/CONT	CAR-P12/CONT	CAR-P3/CAR-P12	CAR-P3/CONT	CAR-P12/CONT	CAR-P3/CAR-P12
GABA_B_1d	1.6	ND	2.1	2.2	ND	2.2
GAD65	2.1	1.8	1.2	2.5	1.4	1.8
A1 aden. rec.	2.2	ND	2.0	1.55	ND	1.64
Pre-proenkephalin	2.0	ND	1.5	1.5	ND	1.9
σ rec.	3.2	ND	2.9	2.3	ND	2.5
NPYR6	3.5	ND	2.7	2.8	ND	2.8
γ-tachikinin	2.1	ND	2.3	2.7	ND	3.1
δ-preporotachykinin	2.2	1.5	1.5	3.1	1.8	1.7
α2b adr. rec.	2.0	1.6	1.2	3.0	1.7	1.8
β3 adr. rec.	2.2	1.7	1.3	3.0	1.7	1.8
5-HT5a	2.0	ND	1.8	1.6	ND	1.2
CCKR-1	2.2	1.8	1.2	3.4	2.3	1.5
IL-10	4.3	1.6	2.6	2.0	1.4	1.4
NR2B	2.2	3.7	0.6	2.4	5.0	0.5
SstR2	2.2	3.2	0.7	3.9	5.3	0.7

In addition to bilateral changes, comparison of the CONT and CAR-P3 groups revealed neonatal CAR injection-induced up-regulation (by 1.9–3.2 fold) of seven genes (coding for GABA_B_1f, GABA_B_1b, 5-HT1b serotonergic, NtsR1 neurotensin and H1 histamine receptors, GAG67, and preprocholecystokinin) confined to the left LDH ipsilateral to the neonatal injury (Table 2). These changes were undetectable in CAR-P12 rats, indicating their CAR-P3 specificity.

**Table 2:** Genes with significantly altered basal levels in the left LDH ipsilateral to the neonatally-injured hindpaw in CAR-P3 as compared to their expressions in the corresponding LDH of CONT and CAR-P12 animals [one-way ANOVAs, (with the Benjamini and Hochberg's FDR correction procedure set at the *P*-value cutoff of 0.05) followed by Tukey's post-hoc tests (with the same *P*-cutoff value) in the implementation of GeneSpring GX software (Agilent, Palo Alto, CA).)]. Differences in gene expression are presented as ratios between CAR-P3 and CONT, CAR-P12 and CONT, and CAR-P3 and CAR-P12 groups. Statistically insignificant differences are marked as 'ND' The comparisons are based on the microarray profiling that employed 5 slides for comparison of CONT and CAR-P3 samples and another 5 slides for comparison of CONT and CAR-P12 samples.

**Left LDH (ipsilateral to the site of neonatal injury)**							
Genes	CAR-P3/CONT	CAR-P12/CONT	CAR-P3/CAR-P12	Genes	CAR-P3/CONT	CAR-P12/CONT	CAR-P3/CAR-P12

GABA_B_1b	1.9	ND	1.7	NtsR1	2.1	ND	1.6
GABA_B_1f	3.2	ND	2.0	5-HT1b	2.0	ND	1.8
GAD67	2.0	ND	1.4	Preprocholecystokinin	2.2	ND	2.0
H1 hist. rec.	3.2	ND	1.7				

#### Adult inflammation-induced Changes in Gene Expression in the LDH Ipsilateral to the Neonatally-injured Hindpaw

In the left LDH ipsilateral to the site of neonatal injury in CAR-P3 rats, twenty-six genes showed side-selective adult inflammation-induced up-regulation (ranging from 1.4 to 6.1 fold) compared to their expressions in the corresponding LDH of CONT animals with similarly inflamed left hindpaw (Table 3). These included genes coding for: NR1-4a and NR3B subunits of NMDA receptor, GluRD and GluR6 subunits of AMPA receptor, GABA_A_α2 and GABA_A_α4, subunits of GABA_A _receptor, GABA_B_1b receptor, 5-HT1a, 5-HT1d, 5-HT1e, 5-HT1f, 5-HT2c, 5-HT4, 5-HT5a, and 5-HT6 serotonergic receptors, calcitonin gene-related polypeptide-2 (CALK-2), neurotrophin 3 (NT-3), TrkA and TrkC neurotrophin receptors, IL-1α, IL-6, IL-12A, IL-15, IL-20, and IL-24 interleukins, and IL-12 β1 interleukin receptor. No significant changes in these genes were observed in the CAR-P12 group (Table 3), indicating that the aforementioned alterations in gene expression were CAR-P3-specific. Additionally, thirteen genes exhibited similarly ipsilateral up-regulations in both CAR-P3 and CAR-P12 rats, but the increases in the CAR-P3 group were significantly greater (by 1.1 – 2.4 fold) than those in the CAR-P12 group (Table 3). These genes encode: NR1-1b, NR2A, and NR2D subunits of NMDA receptor, mGluR1a metabotropic receptor, GABA_A_α5 and GABA_A_α6, subunits of GABA_A _receptor, GABA_B_1c receptor, 5-HT5b serotonergic receptor, calcitonin gene-related peptide receptor (CGRPR), NT-4 neurotrophin, and IL-5 interleukin. Finally, one gene (encoding NK2 tachykinin receptor), while also being up-regulated in the CAR-P3 and CAR-12 groups, showed a significantly weaker increase in CAR-P3 rats as compared to that in CAR-P12 animals (by 0.7 fold).

**Table 3:** Genes with significant adult inflammation-induced alterations in their expressions in the left LDH (ipsilateral to the CFA-inflamed neonatally-injured hindpaw) in CAR-P3 rats as compared to those in the left LDH of CONT and CAR-P12 animals after inflammation of the corresponding paw [one-way ANOVAs, (with the Benjamini and Hochberg's FDR correction procedure set at the *P*-value cutoff of 0.05) followed by Tukey's post-hoc tests (with the same *P*-cutoff value) in the implementation of GeneSpring GX software (Agilent, Palo Alto, CA).)]. Differences in gene expression are presented as ratios between CAR-P3 and CONT, CAR-P12 and CONT, and CAR-P3 and CAR-P12 groups. Statistically insignificant differences are marked as 'ND' The comparisons are based on the microarray profiling that employed 5 slides for comparison of CONT and CAR-P3 samples and another 5 slides for comparison of CONT and CAR-P12 samples.

**Left LDH (ipsilateral to the site of neonatal injury)**							
Genes	CAR-P3/CONT	CAR-P12/CONT	CAR-P3/CAR-P12	Genes	CAR-P3/CONT	CAR-P12/CONT	CAR-P3/CAR-P12

5-HT1a	3.3	ND	2.4	GABA_A_α2	2.0	ND	2.0
5-HT1d	1.9	ND	2.1	GABA_A_α4	1.8	ND	2.0
5-HT1e	1.6	ND	1.6	GABA_A_α5	1.9	1.4	1.4
5-HT1f	1.8	ND	2.0	GABA_A_α6	2.9	1.7	1.7
5-HT2c	1.4	ND	1.7	GABA_B_1b	2.6	ND	2.4
5-HT4	1.9	ND	2.4	GABA_B_1c	2.2	1.5	1.5
5-HT5a	3.0	ND	2.3	NR1-1b	3.2	2.4	1.3
5-HT5b	3.1	2.1	1.5	NR1-4a	2.2	ND	2.0
5-HT6	1.7	ND	1.7	NR2A	2.1	1.5	1.4
IL-1α	1.6	ND	1.6	NR2D	2.3	1.5	1.5
IL-5	2.5	1.6	1.6	NR3B	1.6	ND	2.0
IL-6	1.8	ND	2.2	GluRD	2.0	ND	2.0
IL-12A	1.7	ND	1.3	GluR6	2.1	ND	1.7
IL-12R β1	6.1	ND	4.3	mGluR1a	2.5	1.4	1.8
IL-15	2.4	ND	2.0	CGRPR	2.6	1.5	2.4
IL-20	3.4	ND	2.4	NT-4	2.0	1.2	1.6
IL-24	3.5	ND	3.0	CALK2	2.5	ND	1.9
TrkA	2.3	ND	2.0	VGF	2.6	ND	1.4
TrkC	1.5	ND	1.4	NK2	1.5	2.2	0.7

Among the genes examined in this study, only one (encoding σ receptors) showed significant bilateral changes in its expression in CAR-P3 rats (but not in CAR-P12 animals) after adult inflammation of neonatally-injured hindpaw (left side: CAR-P3/CONT = 3.7 and CAR-P3/CAR-P12 = 2.6; right side: CAR-P3/CONT = 2.8 and CAR-P3/CAR-P12 = 1.9).

### Verification of the Microarray-identified Alterations in Gene Expression by Real-time RT-PCR

**Figure 1:**
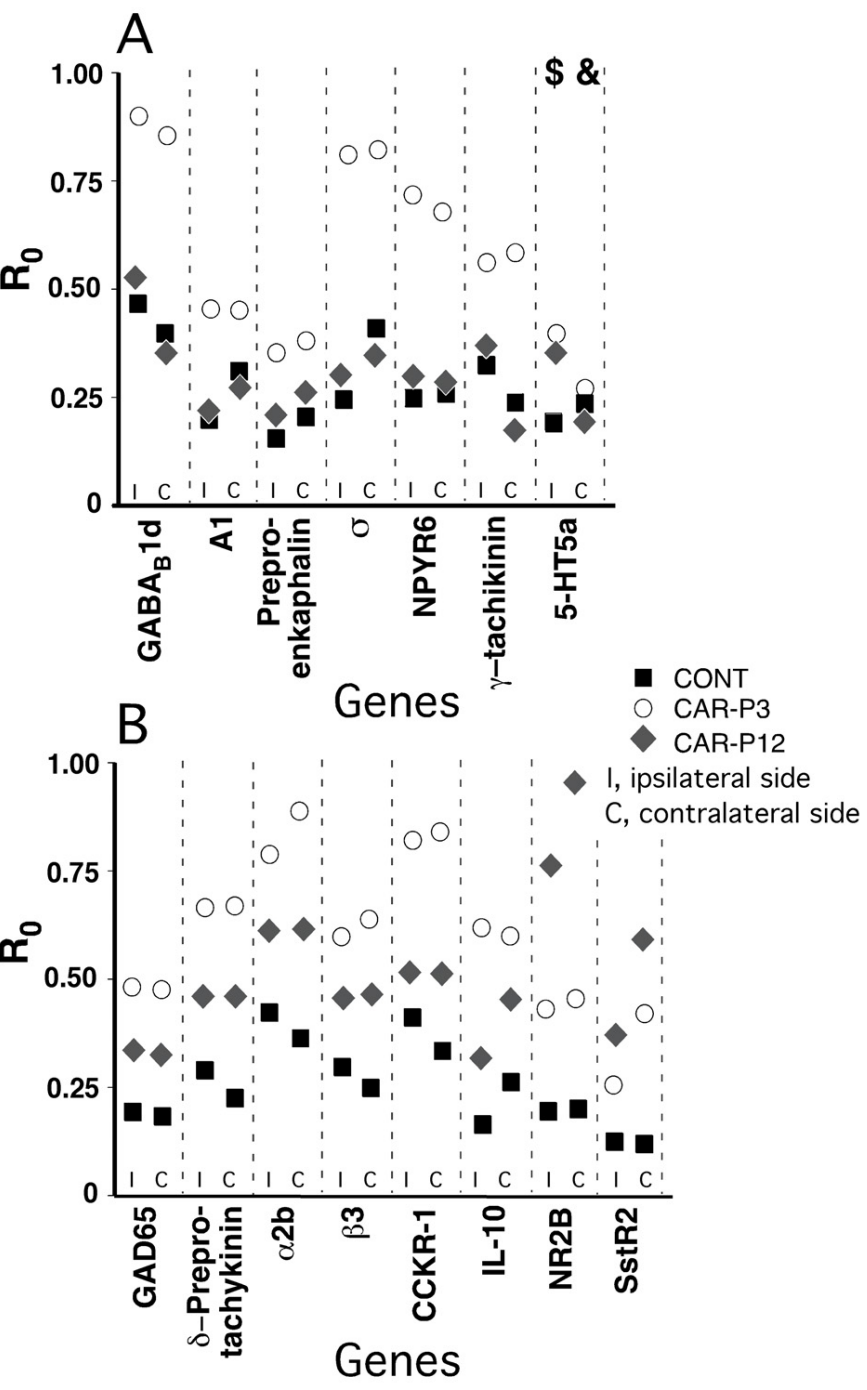
Real-time RT-PCR-determined levels of expression (calculated as R_0 _using β-actin as internal control) for genes identified by microarray profiling as having significantly altered basal bilateral levels in the LDH of CAR-P3 as compared to their bilateral expressions in the LDH of CONT and CAR-P12 animals (Table 1). **A**, genes, which were identified by microarrays as having similar expressions in CONT and CARP-12 groups, but changed (up-regulated) in CAR-P3 group. **B**, genes, which were identified by microarrays as having differences in expression between CONT and CAR-P3 and CAR-P12 groups as well as between CARP-3 and CAR-P12 groups (both when CAR-P3 > CAR-P12 and CAR-P3 < CAR-P12). The data for the LDH ipsilareal to the neonatally-injured paw and the LDH contralateral to that paw are shown separately. Each data-point represents a mean of 5 animals in a group. The data were analyzed using one-way ANOVAs, (with the Benjamini and Hochberg's FDR correction procedure set at the *P*-value cutoff of 0.05) followed by Tukey's post-hoc tests (with the same *P*-cutoff value) in the implementation of GeneSpring GX software (Agilent, Palo Alto, CA). The presented real-time RT-PCR data support the microarray-based findings except when marked by '$' sign (microarrays suggested CARP-3 expression > CARP12 expression, while RT-PCR showed CAR-P3 expression = CAR-P12 expression) and '&' sign (in contrast to microarrays, no inter-group differences were detected by RT-PCR).

**Figure 2:**
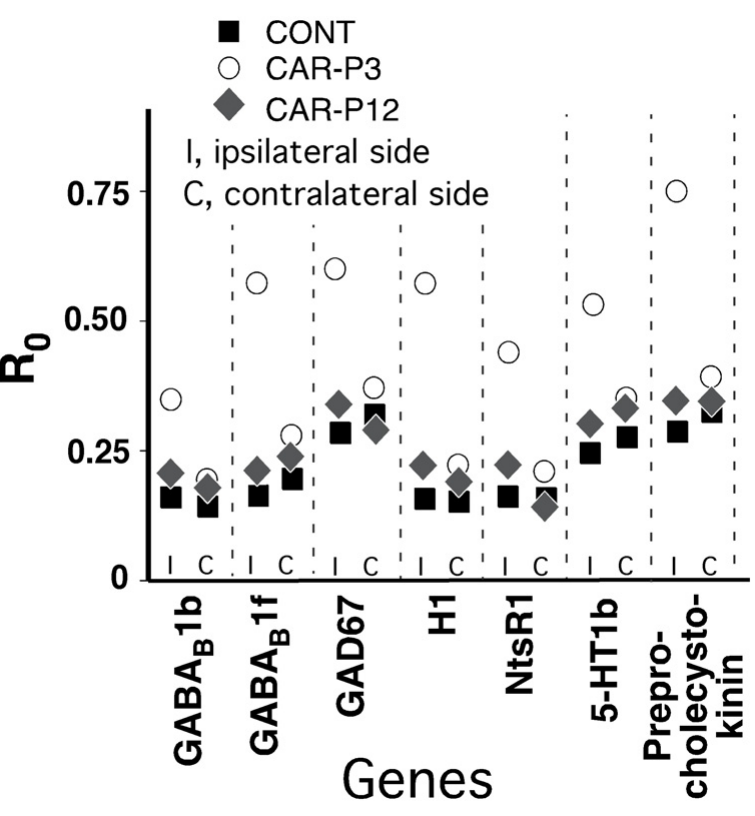
Real-time RT-PCR-determined levels of expression (calculated as R_0 _using β-actin as internal control) for genes identified by having significantly altered basal levels in the left LDH ipsilateral to the neonatally-injured hindpaw in CAR-P3 as compared to their expressions in the corresponding LDH of CONT and CAR-P12 animals (Table 2). The data for the LDH ipsilareal to the neonatally-injured paw and the LDH contralateral to that paw are shown separately. Each data-point represents a mean of 5 animals in a group. The data were analyzed using one-way ANOVAs, (with the Benjamini and Hochberg's FDR correction procedure set at the *P*-value cutoff of 0.05) followed by Tukey's post-hoc tests (with the same *P*-cutoff value) in the implementation of GeneSpring GX software (Agilent, Palo Alto, CA). The presented real-time RT-PCR data support the microarray-based findings.

**Figure 3:**
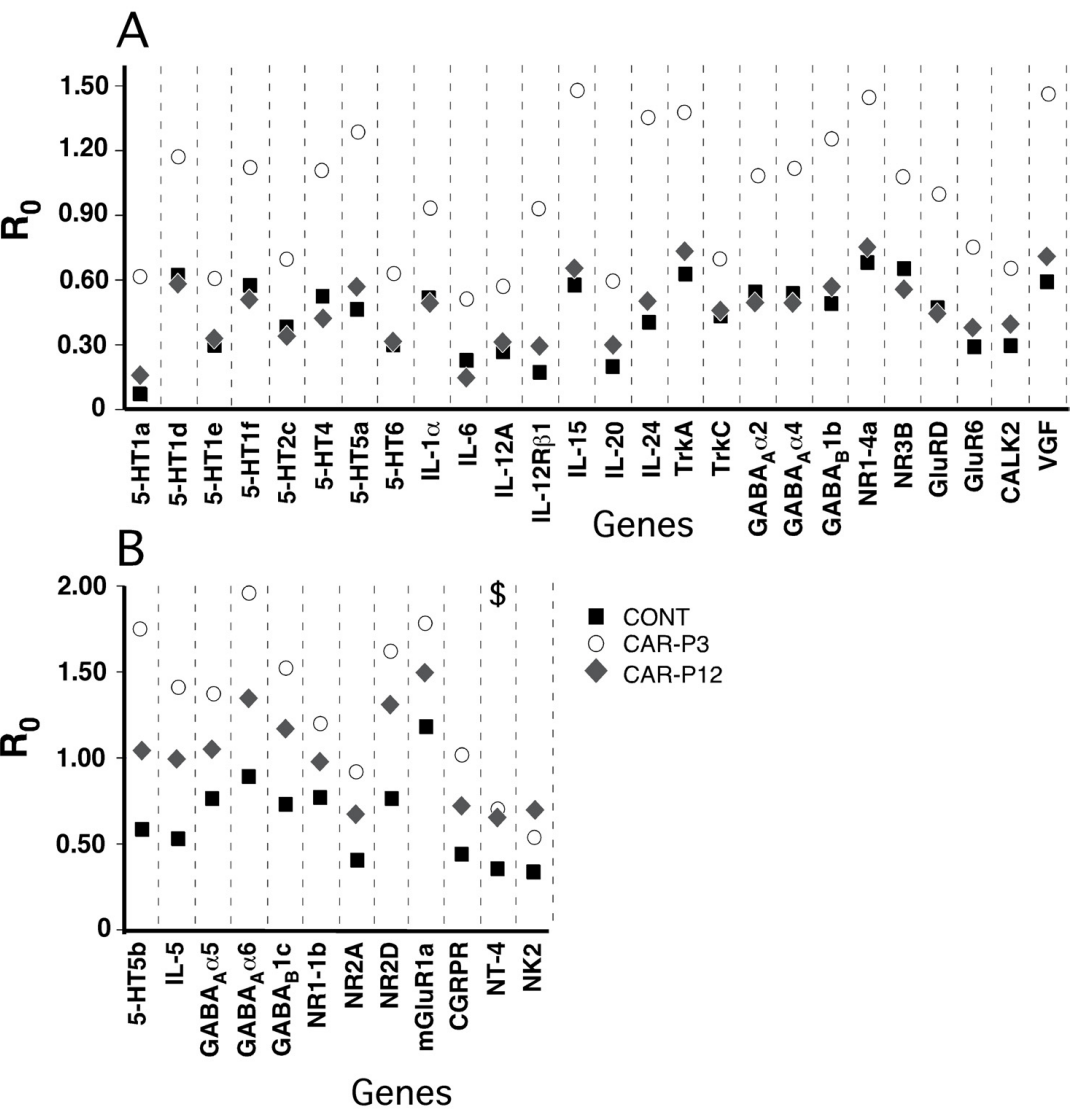
Real-time RT-PCR-determined levels of expression (calculated as R_0 _using β-actin as internal control) for genes identified by microarray profiling as showing significant adult inflammation-induced alterations in their expressions in the left LDH (ipsilateral to the CFA-inflammed neonatally-injured hindpaw) in CAR-P3 rats as compared to those in the left LDH of CONT and CAR-P12 animals after inflammation of the corresponding paw (Table 3). **A**, genes, which were identified by microarrays as having similar expressions in CONT and CARP-12 groups, but changed (up-regulated) in CAR-P3 group. **B**, genes, which were identified by microarrays as having differences in expression between CONT and CAR-P3 and CAR-P12 groups as well as between CARP-3 and CAR-P12 groups (both when CAR-P3 > CAR-P12 and CAR-P3 < CAR-P12). Each data-point represents a mean of 5 animals in a group. The data were analyzed using one-way ANOVAs, (with the Benjamini and Hochberg's FDR correction procedure set at the *P*-value cutoff of 0.05) followed by Tukey's post-hoc tests (with the same *P*-cutoff value) in the implementation of GeneSpring GX software (Agilent, Palo Alto, CA). The presented real-time RT-PCR data support the microarray-based findings except when marked by '$' sign (microarrays suggested CARP-3 expression > CARP12 expression, while RT-PCR showed CAR-P3 expression = CAR-P12 expression).

The real-time RT-PCR analysis supported the findings of the microarray gene profiling, with two exceptions (Figs. 1, 2, 3). First, at the baseline, the expressions of the gene coding for 5-HT5a receptor, although upregulated vs. CONT rats were similar in CAR-P3 and CAR-P12 animals on the side ipsilateral to the neonatally-injured paw. There were also no differences in the expression of this gene between all three groups on the contralateral side (Fig. 1). Second, no differential expression of the gene for NT4 factor was detected between our animal groups after adult inflammation (Fig. 3). Consequently, these effects identified by microarrays were judged to be false.

## Discussion

This study has revealed that CAR injection of a hindpaw in rat pups during neonatal sensitive period leads to abnormal expression levels of multiple genes in the LDH of adult animals, both at the basal and CFA-induced inflammation states. All of the affected genes showed up-regulation, which parallels the earlier observation that peripheral nerve injury largely causes up-regulation of genes encoding signal carrying and modulating proteins in the LDH [10]. Our findings support the notion that regulatory mechanisms of gene expression in the pain-processing circuitry of the spinal cord are vulnerable to long-term alterations by early local noxious insult.

### Bilateral Alterations in Gene Expression in the LDH at Baseline

Among 205 genes profiled in this study, the expression of twelve genes were up-regulated in a bilateral fashion in the LDH of CAR-P3 rats either exclusively or much stronger than that in CAR-P12 animals. These genes encode members of several protein groups, such as GABA synthesis enzymes and receptors, interleukins and their receptors, serotonin, adenosine, σ, NPY, and CCK receptors, and opioid, and tachykinin peptides; yet, each of these groups is represented only by one or two genes suggesting that, at least for the examined genes, the observed changes in expression are diffused, without any group being perceived as a dominant carrier of these changes. However, it is interesting that the translational products of the majority of these genes are most likely to enhance inhibitory processing of nociceptive input in the LDH. These include genes encoding GAD65 GABA synthesis enzyme, anti-inflammatory cytokine, IL-10, and GABA_B_1d, A1 adenosine, α2b and β3 adrenergic, and NPYR6 receptors [11-23]. The remaining genes encode largely pro-nociceptive products, CCKR-1 and σ-receptors, γ-tachykinin, and δ-preprotachikinin [24-26]. Overall, these data are consistent with the previous findings of bimodal inhibitory and facilitatory changes in descending spinal modulation of nociception after tissue injury [27]. Nevertheless, this prevalence of bilateral changes in gene expression promoting anti-nociception points to a possible net increase in the spinal nociception inhibitory drive, which would be a contributing factor to the bilateral hypoalgesia in CAR-P3 rats. An enhanced spinal anti-nociceptive drive is further supported by our recent microarray gene profiling studies in the periaqueductal gray (PAG), which is a major brain center involved in endogenous pain control. These studies showed that the PAG in CAR-P3 animals is characterized by up-regulation of several genes involved in promoting descending inhibition in these animals [28].

It should also be noted that not all alterations in gene expression were stronger in CAR-P3 then in CARP-12 animals. We found two genes that were up-regulated in CAR-P3 group less than in CARP-12 group; one (encoding NR2B subunit of MNDA receptor) is likely pro-nociceptive, while another (encoding SstR2 receptor) is anti-nociceptive [29,30].

### Changes in Gene Expression in the LDH Ipsilateral to Neonatally-Injured Hindpaw at the Baseline and During Adult Inflammation

Without adult inflammation, seven genes showed significant CAR-P3-specific up-regulation in their expression in the LDH ipsilateral to the neonatally-injured hindpaw. Three of these genes code for anti-nociceptive products involved in GABA neurotransmission: GAD 67, and GABA_B_1b and GABA_B_1f receptors [12-14], while the other four genes generate potentially pro-nociceptive products, preprocholecystokinin, and NtsR1 neurotensin, H1 histamine and 5-HT1b serotonergic receptors [26,31-34]. These findings do not support our expectation of strong gene-expression driven baseline inhibition of pain processing in the LDH on the neonatally-injured side. Clearly, more studies are needed to reconcile long-term changes induced specifically in the ipsilateral LDH by neonatal inflammation of a hindpaw with the apparent similarity in baseline pain responsiveness of both hindpaws.

Previously, we suggested [9] that specific localized early noxious insult-induced long-term deficits in the molecular organization of the spinal circuitry processing nociceptive information from the neonatally-injured hindpaw are likely to be most notable during a strong local challenge, such as CFA-induced inflammation of the neonatally-injured paw. Indeed, the present study noted that adult inflammation of the left hindpaw (which had received CAR injections in CAR-P3 and CAR-P12 animals) induced significant up-regulation in expression of thirty-six genes in the ipsilateral LDH of CAR-P3 rats as compared to that in similarly-inflamed CONT animals. These differences were either absent or were significantly less pronounced in CAR-P12 animals. Furthermore, in contrast to the baseline conditions, the adult inflammation-induced changes in multiple members of several large gene groups in our arrays. In particular, eight of the affected genes encode interleukins (IL-1α, IL-5, IL-6, IL-12A, IL-15, IL-20, IL-24) and the interleukin receptor, IL-12 β1, which are pro-inflammatory molecules [35-39]. Another eight genes encode glutamate neurotransmission-related proteins, including metabotropic mGluR1a receptor, subunits of NMDA receptor (NR1-4a, NR1-1b, NR2A, NR2D, NR1-4a) and AMPA glutamate (GluRD and GluR6) receptors, which play mostly pro-nociceptive roles in the LDH [13,40]. The up-regulated subunits of NMDA and AMPA receptors are known to have multiple roles in modulating functionality of these receptors, nevertheless, the overall effect of their up-regulation is likely to increase NMDA and AMPA receptor ligand sensitivity and intracellular signaling [41-46]. The next group of eight genes encodes eight 5-HT serotonergic receptors (5-HT1a, 5-HT1d, 5-HT1e, 5-HT1f, 5-HT2c, 5-HT4, 5-HT5a, and 5-HT6). Unfortunately, the specific outcome of the observed up-regulation of these receptors is difficult to predict, since the actions of most of these receptors have not been tested in spinal cord neurons, and those receptors that have been examined are capable of exerting both pro- and anti-nociceptive actions depending on their cellular position within the spinal dorsal horn [13]. Finally, a group of six genes encodes either subunits of the GABA_A _receptor (GABA_A_α2, GABA_A_α4, GABA_A_α5, and GABA_A_α6 – all subunits enhancing activity of GABA_A _ionic channels; [47]) or GABA_B _receptors (GABA_B_1b and GABA_B_1c). As mentioned earlier, these receptors inhibit neuronal facilitation in the spinal dorsal horn [12-14]. The remaining genes code for CGRP, CGRPR receptor, and CALK-2, which have been suggested to have a pro-nociceptive influence in the LDH [13], and neurotrophin-3, and TrkA and TrkC neurotrophin receptors, which reportedly participate in the neurotrophin-associated maintenance of the central spinal sensitization associated with persistent pain [48-53]. Based on the aforementioned observations, it is reasonable to assume that the abnormal regulation of expression of numerous genes involved in processing nociceptive information in the LDH ipsilateral to the neonatally CAR-injected hindpaw may play a significant role in the enhanced hyperalgesia during inflammation of this paw in adult rats.

Among the examined genes, only one (encoding NK2 receptor) showed stronger adult inflammation-induced expression in animals subjected to neonatal CAR injection outside of the sensitive period as compare to those receiving CAR within this period. Activation of NK2 is known to promote analgesia [54].

Finally, it is interesting that very few genes expressing significant inter-group differences in their levels prior to inflammation showed such differences after inflammation was re-introduced in adult animals. Only GABA_B_1b and σ receptors continued to display significant up-regulation upon adult inflammation of the neonatally-injured paw in the LDH of CAR-P3 rats in the ipsilateral and bilateral manner respectively. Unfortunately, the microarray analysis as performed in this study, does not allow us to discern the reasons why the majority of genes displaying baseline CAR-P3-specific changes failed to show alterations during adult inflammation.

The limited screening of gene expressions described in this paper is just the first step toward understanding the molecular mechanisms involved in long-term alteration in nociception produced by early local noxious insult. Both much wider high throughput profiling studies, including those of proteins, and more focused and detailed investigations, addressing the sites of molecular, alterations on the cellular level and experimental testing of their functional consequences, are needed to fully address this question.

## Methods

### Animals

Adult female and male Sprague-Dawley breeders were purchased from Harlan (Indianapolis, IN) and bred at the University of Maryland Animal Facility. Litters with ≥10 pups were selected for this study. They were divided into three groups. In the CAR-P3 and CAR-P12 groups, the pups received a single injection of 0.25% CAR in sterile 0.9% saline (1 μl/g) in to the plantar surface of their left hindpaw at 9:00 pm on either P3 or P12 respectively. The injection was conducted with a microliter syringe with a 31-gauge needle. Previously, we demonstrated that, at both ages, such an injection induces short-lasting (~48 h) increase in the diameter of the injected paw, accompanied by reduced withdrawal latencies to thermal and mechanical noxious stimulation [8]. Pups in the CONT group received no injections. Except for the hindpaw injection procedure, all rats were subject to the same human intervention involved in routine animal care such as weekly cage cleaning and daily food and water changes, etc. We did not include a brief handling control group since brief handling after birth has been shown not to produce long-term alterations in pain responsivity [8]. A saline-injected control group also was not included since saline injection by itself is a noxious experience and it is beyond the scope of the present study to distinguish between long-term consequences of different types of early noxious insults. After weaning at P21, male pups were housed in groups of three with animals in any given cage receiving the same neonatal treatment. The females were used in different experiments (we understand the importance of extending the present studies to females, which will be done in the future). At P59, the left hindpaws of half of the rats in CONT, CAR-P3 and CAR-P12 groups received an inflammation-inducing injection of CFA (0.05 ml, 1:1 oil/saline emulsion). The rest of the animals received no injections. Twenty-four hours later (at P60), all rats were anesthetized by i.p. injections of Nembutal. The left and right LDH of the L4, L5 levels were dissected out and snap-frozen in liquid nitrogen. All experiments were approved by the University Institutional Animal Care and Use Committee.

### Microarrays

In this part of the study, we used left and right sides of the LDH from ten CAR-P3, ten CAR-12, and ten CONT animals, one half at baseline and another half after CFA-induced inflammation of the neonatally-injured paw per group. For both baseline and inflammation states, each animal belonged to a different litter. For each state, the collected samples were processed in randomly selected CAR-P3/CONT and CAR-P12/CONT pairs per microarray slide [5 pairs each; 10 pairs for both comparison groups × 2 (left and right) = 20 microarray slides total/inflammation state].

For the analysis, the total sample RNA was isolated with TRIzol protocol (Invitrogen, Carsbad, CA). The RNA yield was determined using a DU 640 Spectrophotometer (Beckman, Coulter, Fullerton, CA). The 260/280 nm ratios of the samples were 1.8. The RNA isolated from a specific pairs of samples (5 μg RNA per sample) was reverse transcribed utilizing a 3DNA Array Detection Kit (Genesphere, Hartfield, PA); with primers containing different capture sequences for CONT and experimental (CAR-P3 and CARP-12] tissue. The resultant cDNA was then hybridized to oligonucleotide microarrays custom designed and printed for us by TeleChem International (Sunnyvale, CA). The sequences of the chosen mRNAs were obtained from the Gene Bank  with their unique regions identified with the help of the 'BLAST-2 Sequences' and 'Multiple Alignment' websites ( and  respectively). The same two websites were used for the verification of the uniqueness of the oligoprobe sequences selected by TeleChem International. Probes for four internal standards, glyceradehyde-3-phosphate-dehydrogenase (GAPDH), β-actin, ubiquitin, and βIII-tubulin, were also included in the microarrays. The oligonicleotide probes were printed with the TeleChem's advanced Stealth technology on SuperAldehyde substrate coated-glass slides in quadruplicates, with each of the four sets of probes occupying a different quadrant of the slide microarray matrix. The cDNA hybridization was performed at 55°C for 16 h in 2 × SDS-Based Hybridization Buffer (3DNA Array Detection Kit, Genesphere, Hartfield, PA). The hybridization was followed by washing with 2 × SSC and 0.2% SDS at 42°C, 2 × SSC at room temperature, and 0.2 × SSC at room temperature, 10 min each. Washed slides were air-dried for 30 sec. For fluorescent labeling of microarray-bound cDNA, the slides were incubated for 3 h at 65°C with a Capture Reagent Hybridization Mixture from a 3DNA Array Detection Kit (Genesphere Inc, Hatfield, PA). In this mixture, Alexa 546 fluorochrome-incorporating dendrimers contained single-stranded arms complimentary to the 'capture' sequences used in the reverse transcription of RNA from CONT samples, while Alexa 647 fluorochrome-incorporating dendrimers contained arms complimentary to the capture sequences used in reverse transcription of RNA from CAR-P3 and CARP-12 samples. The labeling reaction was terminated by washing the microarray slides at room temperature for 10 min with 0.005% Triton in 2 × SSC and for another 10 min in 0.2 × SSC. After that, the slides were air-dried for 30 sec. The processed microarray slides were scanned on a GenePix 4100A scanner (Axon Instrument, Union City, CA) with the laser excitation at 532 nm [emission filter 575DF35 (green); photomultiplier voltage 550] for Alexa 546 (control samples) and the laser excitation at 635 nm [emission filter 670DF40 (red); photomultiplier voltage 695] for Alexa 647 (experimental samples). The densitometry was performed with GenePix Pro 4.1 (Axon Instruments, Union City, CA). Background was subtracted using the 'Local Background Correction' procedure and quality control was achieved by 'Quality Control' feature of the GenePix software. Since our microarray slides generated four spots per gene, the median intensity of these quadruplicates was calculated. For each array, the data were normalized with Acuity 3.1 (Axon Instruments, Union City, CA) by applying locally weighted scatterplot smoothing (LOWESS) transformation that utilizes all genes in the microarray [55] and by the internal standards, GAPDH, beta-actin, ubiquitin and βIII-tubulin [56]. LOWESS normalization eliminated non-linear die distortion of the microarray data [55]. The statistical analysis was conducted separately for each inflammation state of the adult animals and the left and right sides as described in [28]. For this analysis, the data from the CONT samples were adjusted to produce a uniform value of 1. Then, the levels of the gene expressions between CAR-P3, CAR-P12, and CONT groups were compared employing one-way ANOVAs (with the false discovery rate (FDR) multiple testing error correction procedure of Benjamini and Hochberg [57] set at the *P*-value cutoff of 0.05) followed by Tukey's post-hoc tests (with the same *P*-cutoff value) in the implementation of GeneSpring GX software (Agilent, Palo Alto, CA).

### Real-Time RT-PCR

For the verification of the changes in gene expression suggested by the microarray analysis, real-time RT-PCR was conducted in 5 samples per relevant group, each obtained from an animal belonging to a different litter. Also, none of these litters were used in the above-described microarray analysis. All the baseline changes were re-examined bilaterally. The changes induced by CFA-based inflammation were re-examined only ipsilaterally. β-actin was employed as the endogenous control [28]. For each assay, the total RNA was isolated as described for microarray analysis, and then treated with Amplification Grade Deoxyribonuclease I (1 u/μg RNA; Invitrogen, Carlsbad, CA) for 15 min at 25°C. Reverse transcription was performed using Omniscript RT kit (Qiagen, Vanencia, CA) using random hexamers as primers. Real-time PCR utilized TaqMan assay, which requires two primers and a fluorescence-labeled probe for cDNA amplification and visualization. All TaqMan PCR primers and probes were designed using Primer Express 1.5a (Applied Biosystems, Foster City, CA) and custom-synthesized by Applied Biosystems (Foster City, CA). The PCR reactions were carried out on an ABI Prism 7000 Sequence Detector (Applied Biosystems, Foster City, CA). All PCR reactions were run in a monoplex mode – amplification of one gene per reaction well in a PCR plate. Each reaction included sample cDNA (1 μl), the reverse primers (100–500 nM each) and probes (50–100 nM) for the gene of interest, TaqMan Universal Master Mix (25 μl, Applied Biosystems) and deionized water that brought the total volume to 50 μl. The cycles were run at 95°C/10 min, 95°C/15 sec × 45, and 60°C/1 min. All assays were performed in triplicates. The normalization to internal standards and expression of the resultant data was calculated using amplification plot method of Pierson et al. [58] implemented in DART-PCR Excel workbook . In this method amplification efficiency for both target and control genes were calculated from raw data around the midpoint of the transformed signal range, which is more accurate than when derived from an external standard curve [59]. The average efficiency from all (experimental and control) runs of a given gene was then calculated (since this improves accuracy as compared to the use of individual run efficiencies; [58]). The obtained efficiency is used in generating Starting Fluorescence Value for a given gene in a sample (R_0_), which is proportional to the starting template quantity. Finally, R_0 _for the target gene is normalized to R_0 _of the internal control from the same sample [R_0(sample) _= R_0(target)_/R_0(control)_]. To allow inter-plate comparison, the R_0(sample) _is further normalized to R_0(standard) _generated for the same target gene and internal control in the wells with the "standard" cDNA run on the same plate with the experimental samples [R_0(sample 2 × normalized) _= R_0(sample)_/R_0(standard)_]. The 'standard' cDNA for the entire study was generated from a 'standard tissue homogenate' that was pooled from the LDH tissue of 10 rats dedicated for this purpose. To assess possible contamination with chromosomal DNA, several RNA samples were processed for PCR with β-actin probe and primers without the RT step. No amplification products were detected.

The statistical analyses of samples from the left and right sides and from different inflammation states of adult animals were performed separately. These analyses include one-way ANOVAs (with the Benjamini and Hochberg's FDR correction procedure set at the *P*-value cutoff of 0.05) followed by Tukey's post-hoc tests (with the same *P*-cutoff value) in the implementation of GeneSpring GX software (Agilent, Palo Alto, CA).

## Competing interests

The author(s) declare that they have no competing interests.
